# Distributed Fiber Optic Sensing for Fracture Geometry Inversion Using All Time Steps Data

**DOI:** 10.3390/s25144290

**Published:** 2025-07-09

**Authors:** Shaohua You, Geyitian Feng, Xiaojun Qian, Qinzhuo Liao, Zhengting Yan, Shuqi Sun, Xu Liu, Shirish Patil

**Affiliations:** 1 National Key Laboratory of Petroleum Resources and Engineering, China University of Petroleum-Beijing, Beijing 102249, China; 2023310131@student.cup.edu.cn (S.Y.); 2024210284@student.cup.edu.cn (G.F.); qianxiaojun@student.cup.edu.cn (X.Q.); 2024310148@student.cup.edu.cn (Z.Y.); 2023210279@student.cup.edu.cn (S.S.); 2 College of Petroleum Engineering & Geosciences, King Fahd University of Petroleum and Minerals, Dhahran 31261, Saudi Arabia; liu.xu@kfupm.edu.sa (X.L.); patil@kfupm.edu.sa (S.P.)

**Keywords:** distributed optic fiber, hydraulic fracturing, wellbore monitoring, fracture geometry, inversion method

## Abstract

As an advanced real-time monitoring technique, optic fiber downhole sensing has been widely applied in monitoring fracture propagation during hydraulic fracturing. However, existing fracture shape inversion methods face two main challenges: firstly, traditional methods struggle to accurately capture the dynamic changes in strain rate and fracture shape during the propagation process, and secondly, they are computationally expensive. To address these issues, this study proposes a full-time-step fitting inversion method. By precisely fitting all time steps of fracture propagation, this method effectively overcomes the shape deviation problems often encountered in traditional methods and significantly reduces computational costs. Compared to conventional single-time-step inversion methods, our approach not only provides a more accurate representation of the spatiotemporal dynamics of fracture propagation but also avoids the risk of significant errors in fracture shape reconstruction. Therefore, the proposed inversion method holds substantial practical value and significance in fracture monitoring and sensing for oil and gas fields.

## 1. Introduction

Distributed fiber optic sensing is becoming increasingly important in the oil and gas development industry, especially when dealing with unconventional reservoirs that are low in porosity, low in permeability, dense, and highly heterogeneous [1,2]. Hydraulic fracturing reservoir stimulation technology is commonly used to enhance the seepage capacity of unconventional oil reservoir formations [3]. During hydraulic fracturing, a large volume of fluid is injected into the formation and retained in the lower layers under pressure. When the pressure exceeds the rock’s fracture pressure, the fluid opens the reservoir, forming a complex fracture network with both artificial and natural fractures. This fracture network, supported by proppants, creates permanent flow channels, increasing the contact area between the oil and gas well and the reservoir, which is beneficial for subsequent extraction. After implementing fracturing stimulation technology, the fracturing effect is typically evaluated. Understanding the hydraulic fracturing fracture propagation mechanism is crucial for controlling fracture extension and optimizing fracturing results [4]. This knowledge is essential for managing and optimizing the production of horizontal wells and guiding subsequent fracturing optimization designs. Due to the complex stress state of unconventional reservoirs, strong rock mechanics anisotropy and heterogeneity, interference between artificial and natural fractures, and unclear monitoring [5,6,7,8,9], traditional hydraulic fracturing monitoring and diagnostic methods often struggle to evaluate fracturing effects in a timely and effective manner [10]. As a result, traditional hydraulic fracturing methods can no longer meet the current extraction needs of unconventional reservoirs. Therefore, improving fracturing effects and rapidly and efficiently diagnosing fracturing outcomes have become important topics in modern oil and gas reservoir development [11].

In this context, distributed fiber optic fracturing monitoring technology has emerged as a new method for hydraulic fracturing monitoring [12]. Due to its high sensitivity, real-time monitoring, and resistance to high temperature and pressure, distributed fiber optic fracturing monitoring technology has gradually become an important tool for monitoring fracture propagation during hydraulic fracturing [13]. Compared to traditional downhole monitoring methods, distributed fiber optic sensing offers the advantage of full-process, full-space monitoring. It can cover the entire wellbore in the vertical direction and provide continuous spatial resolution along the entire fiber path, enabling real-time downhole monitoring under various conditions such as production and injection [14]. This capability allows for accurate monitoring of downhole temperature and acoustic changes [15,16,17], real-time collection of downhole data, and analysis of fracture propagation and distribution, providing data support for subsequent optimization designs and reducing environmental impacts during fracturing operations. Additionally, distributed fiber optic sensing, lacking electronic components, is immune to electromagnetic interference, allowing it to function normally in complex downhole environments. Its resistance to high temperature and pressure also gives it greater durability compared to traditional downhole monitoring tools. These advantages make distributed fiber optic fracturing monitoring technology an important and indispensable tool in the development of unconventional oil and gas reservoirs, especially under complex geological conditions.

Distributed fiber optic fracturing monitoring technology can be categorized into two types based on fiber arrangement: in-well and near-well monitoring. This study primarily employs near-well distributed fiber optic monitoring, where fiber optic sensors are arranged in a monitoring well parallel to the fracturing well, with the fiber laid along the depth direction of the fracturing well to monitor fracture propagation in real-time during the fracturing process. In recent years, with the increasing application of distributed fiber optics in oilfields, there have been new developments in monitoring and interpreting artificial fracture initiation. In addition to traditional distributed acoustic sensing (DAS) and distributed temperature sensing, low-frequency distributed acoustic sensing (LF-DAS) and Rayleigh frequency shift distributed strain sensing can also be used to interpret fracture initiation in horizontal well multi-stage fracturing and perform fracturing diagnostic evaluations [18]. This study primarily utilizes near-well fiber optic low-frequency vibration monitoring. Near-well fiber optic LF-DAS utilizes the sensing function of fiber optics by collecting DAS monitoring data and extracting low-frequency data to analyze and interpret various aspects of hydraulic fracturing [19]. Specifically, fiber optic sensors are distributed along the trajectory of the monitoring well. During fracturing operations, as fractures propagate and expand, the rock layers release seismic or vibration waves of specific frequencies due to stress changes. Since these vibration signals during fracture propagation are primarily concentrated in the low-frequency range, fiber optics, as a sensing medium, can efficiently capture these low-frequency signals and transmit them to the surface monitoring system through the optical waveguide properties of the fiber. The monitoring system processes the received signals in the time-space domain and performs spectral analysis, thereby accurately reflecting key information such as fracture propagation paths, widths, and depths.

Traditional inversion methods involve obtaining field fiber optic data, constructing Green’s functions using the three-dimensional displacement discontinuity method, and then solving linear equations using the least squares method. Through Markov Chain Monte Carlo simulations [20], fracture width samples are generated from the target distribution of LF-DAS strain data, quantifying uncertainties related to the inverted width. In simple terms, traditional inversion techniques first acquire LF-DAS fiber optic signal diagrams from the field; then, based on fiber optic strain response characteristics, generate LF-DAS waterfall diagrams; by comparing the differences between the two generated signal waterfall diagrams, iterative calculations are performed to make the latter’s characteristics approach the field signal waterfall diagram. Finally, the fracture parameters corresponding to the iteratively calculated results are compared with the actual fracture parameters formed during fracturing to further verify the model’s accuracy. However, this method has certain limitations. In complex downhole environments, collected data are often affected by various noise and interference, leading to low-quality raw data that do not accurately reflect the collected downhole data, thereby affecting inversion accuracy. Although traditional inversion methods can generate certain fracture parameters, they may not accurately reflect the actual fracture propagation process due to reliance on fiber optic strain response characteristics to generate signal waterfall diagrams. This approach overlooks the hydraulic fracturing fracture propagation mechanism, which is key to controlling fracturing behavior and optimizing fracturing results [21]. Signal diagrams generated based on strain response characteristics may differ significantly from actual fracture propagation mechanisms, leading to deviations in inverted parameters such as fracture width, length, and morphology, and failing to truly reflect the real development of fracturing fractures. Therefore, a more fracture propagation-conforming distributed fiber optic inversion interpretation method is needed.

This paper introduces an innovative distributed fiber optic near-wellbore fracturing monitoring and fracture inversion technology. The technology achieves high-precision inversion of fracture parameters through the following key steps, enabling visual characterization of critical fracture information [22]. Ultimately, it provides a foundation for efficient evaluation of hydraulic fracturing performance in oil and gas wells and subsequent optimization design. First, a distributed fiber optic near-wellbore monitoring system is deployed on-site in the oil field to acquire raw DAS data, from which LF-DAS data are extracted. Due to the complex downhole environment, the raw data often contains various interference signals, such as equipment vibrations, fluid impacts, and dynamic changes in the formation. To address this, efficient filtering and denoising algorithms are introduced to preprocess the raw data, removing downhole noise interference and retaining low-frequency valid signals during the fracture propagation process [23]. After data preprocessing, clear and accurate LF-DAS acoustic vibration waterfall plots are generated. These plots intuitively display the changes in vibration signals at various spatiotemporal locations during fracture propagation, providing reliable data support for subsequent fracture inversion analysis. Compared to traditional inversion methods that solely rely on fiber optic strain response characteristics to generate waterfall plots, this approach aims to accurately and reliably characterize the dynamic propagation of fractures during hydraulic fracturing. To efficiently obtain the propagation process and fracture network morphology of hydraulic fracturing fractures [24,25,26], complex problems are simplified by investigating analytical models and solutions [27,28], such as the Khristianovich-Geertsma-De Klerk (KGD), Perkins-Kern (PK), and Perkins-Kern-Nordgren (PKN) models [29,30,31], in combination with actual oil field conditions. Ultimately, the PKN fracture model is innovatively introduced to describe fracture propagation during the hydraulic fracturing process. By introducing the full-time-step PKN model for inversion, dynamic simulation of the entire hydraulic fracturing process is achieved, considering all stages of fracture propagation. This model can accurately predict fracture morphology, dimensions, and conductivity, providing fundamental data for hydraulic fracturing design and subsequent optimization. The full-time-step PKN model accounts for fluid leak-off and proppant transport processes within the fracture, making fracture propagation analysis more precise and capable of handling complex geological conditions, thereby broadening its application range. Parameter sensitivity analysis is conducted to examine key factors influencing fracture propagation and fiber optic strain response, such as in situ stress distribution, rock physical properties, fracture geometry, and injected fluid characteristics. Results obtained from the forward model are used to predict physical fracture parameters (e.g., fracture width, height) through data fitting methods [32], and the model is optimized using regularization techniques to further reduce the impact of multiple solutions and uncertainties during inversion. By constructing models of adjacent fiber optics and fractures, key signal features, such as frequency, amplitude, and phase, are extracted from DAS data based on the established fracture propagation model and fiber optic strain response mechanism. Finally, inversion is performed using the full-time-step PKN model.

Through the application of the above technical methods, the inverted fracture parameters closely match the actual fracture morphology formed during the fracturing process, accurately reflecting the dynamic patterns of fracture propagation during fracturing operations. This inversion method not only enhances the accuracy of fracturing effectiveness evaluation but also provides data support for fracturing design optimization, demonstrating the broad application prospects of distributed fiber optic near-wellbore monitoring technology in unconventional oil and gas reservoir development [33]. In particular, this study focuses on improving the interpretation of strain rate signals acquired by DAS systems, enabling more effective use of fiber optic sensing in field-scale applications. Although the method is demonstrated in the context of hydraulic fracturing, the inversion framework can be extended to other sensing-driven domains such as geotechnical monitoring, carbon capture and storage, and civil infrastructure.

## 2. Methods

Traditional inversion methods typically involve obtaining raw Distributed Acoustic Sensing (DAS) data from the oilfield, extracting LF-DAS data to generate LF-DAS fiber optic signal plots, and creating LF-DAS waterfall diagrams based on fiber optic strain response characteristics. It is noted that since DAS measures dynamic strain rate rather than temperature, thermal effects do not significantly affect the sensing response or inversion results, in contrast to DTS systems. By comparing these generated signal plots and iteratively adjusting calculations to align the features of the latter with the field data, inversion results are derived. However, this approach has certain limitations. Traditional inversion methods rely on signal plots generated from fiber optic strain response characteristics, which may not accurately reflect the actual fracture features. To overcome this limitation, the present method innovatively introduces the PKN fracture model to describe fracture propagation during hydraulic fracturing. By constructing models of PKN fractures and adjacent wellbore fiber optics, and combining fiber optic strain response mechanisms, key signal features from DAS data are extracted to calculate fiber optic strain. Finally, inversion is performed using the full-time-step PKN model as a constraint. This innovative approach enables the final inversion results to closely match the actual fracture parameters formed during the fracturing process. This method enhances the accuracy of fracture parameter inversion, providing a solid foundation for efficient fracturing effect evaluation and subsequent optimization design.

### 2.1. Perkins-Kern-Nordgren Model

The PKN model is a classical model used to describe fracture propagation during hydraulic fracturing. It considers the entire process of fracture extension, accurately simulating the evolution of hydraulic fractures. Additionally, constraining the model with the full-time-step PKN model effectively reduces inversion uncertainties and enhances interpretive accuracy.

The basic assumptions of the full-time-step PKN model are: (a) The hydraulic fracture has a constant height along its extension direction; (b) The fracture is in a plane strain condition within a vertical plane; (c) The fracture toughness does not affect the geometric deformation of the fracture; (d) The fracturing fluid inside the fracture is a Newtonian fluid; (e) The rock matrix adheres to elastic theory [34].

The continuity equation for the fracturing fluid within the fracture is:

(1) $$\frac{\partial q}{\partial x}+q_{1}+\frac{\partial A}{\partial t}=0,0\leq x\leq L$$

where *q* represents the flow rate through the cross-section of the fracture, *q_l_* enotes the fluid loss rate per unit length of the fracture, and *A* signifies the cross-sectional area of the fracture.

The control equation for hydraulic fracture propagation is [34]:

(2) $$\frac{G}{64(1-\nu )\mu h}\frac{\partial^{2}w^{4}}{\partial x^{2}}=\frac{8c_{l}}{\pi \sqrt{t-t_{0}(x)}}+\frac{\partial w}{\partial t}$$

where *w* is the crack opening, *μ* is the dynamic viscosity coefficient of the fracturing fluid, *G* is the rock’s shear modulus, and *v* is the rock’s Poisson’s ratio. The initial conditions for the above equation are [34]:

(3) w(x,0)=0

The boundary condition is [34]:

(4) w(x,t)=0,    x≥L(t)

(5) $${\left(\frac{\partial w^{4}}{\partial t}\right)}_{x=0}=-\frac{256(1-\nu )\mu }{\pi G}q$$

Using the above initial and boundary conditions, when there is no filtration of fracturing fluid in the crack, the crack length is [35]:

(6) $$L=0.68{\left[\frac{Gq^{3}}{(1-\nu )\mu h^{4}}\right]}^{\frac{1}{5}}t^{\frac{4}{5}}$$

Fracture width:

(7) $$w_{0}=2.5{\left[\frac{(1-\nu )\mu q^{2}}{Gh}\right]}^{\frac{1}{5}}t^{\frac{1}{5}}$$

The pressure of the fracturing fluid at the injection wellbore [35]:

(8) $$p_{0}=2.5{\left[\frac{G^{4}\mu q^{2}}{{\left(1-\nu \right)}^{4}h^{6}}\right]}^{\frac{1}{5}}t^{\frac{1}{5}}$$

Although the PKN model is well-established, we provide a detailed formulation here because it forms the foundation of our fiber optic forward model. The fracture geometry computed from the PKN model determines the spatial and temporal distribution of strain along the fiber, which is used to generate synthetic DAS signals. These signals are then compared with field data in the inversion process. Thus, a clear and complete presentation of the PKN model is necessary to support the simulation and inversion framework.

### 2.2. Near-Wellbore Optic Fiber Strain Response

**Figure 1:**
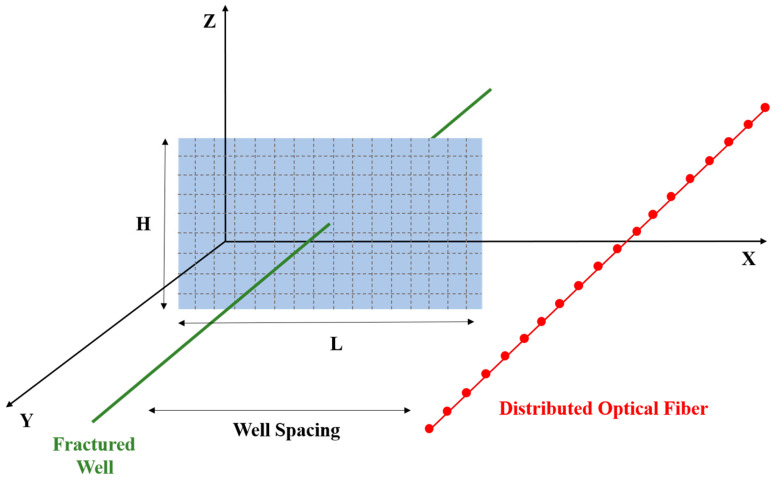
Fracture propagation model and fiber strain model.

After constructing the PKN fracture model and accurately predicting physical parameters such as fracture morphology, dimensions, and conductivity, a near-wellbore fiber optic model is developed. To capture the measurable response of fiber optics to fracture propagation, we establish a near-wellbore fiber optic strain response model. In this model, the fracture geometry predicted by the PKN framework is used to calculate the stress and deformation field in the surrounding formation. Assuming perfect mechanical coupling between the fiber and formation, and considering axial deformation only, the model computes the fiber’s axial displacement based on the displacement discontinuity method. Strain and strain rate are then derived as observable quantities that correspond to DAS signals. The following section presents the mathematical formulation of this model (Figure 1). In this model, the fracture is represented as a rectangular crack with a half-length of L and height H. The monitoring well is positioned at a horizontal distance of *S*_well_ and a vertical distance of *D*_well_ from the fracturing well. The fracture center is set as the coordinate origin, with *x*, *y*, and *z* coordinates corresponding to the fracture length, height, and wellbore axial direction, respectively. The PKN model is employed to calculate the fracture-induced stress field, with fracture element dimensions defined as Δ*x* × Δ*y*.

Although this study is based on numerical simulation, the model configuration closely reflects real DAS deployment scenarios in the field. The fracture geometry, sensor layout, and mechanical coupling assumptions are consistent with those used in laboratory and field experiments. For example, previous works such as Hartog, Wang et al. and Jin et al. have demonstrated that DAS systems can capture axial strain responses caused by fracture deformation. Our fiber optic strain model is designed to replicate these mechanisms and, thus, serves as a validated forward model for synthetic DAS signal generation [36,37]. Fracture opening generates stress and strain in the formation. Fiber optics, well-coupled with the formation, can detect these deformations, thereby diagnosing the dynamic propagation direction of the fracture. Based on the established fracture propagation model and the fiber optic strain response mechanism, key signal features such as frequency, amplitude, and phase are extracted from Distributed Acoustic Sensing (DAS) data.

The basic assumptions of the fiber optic strain model are: (a) Fiber optic displacement is considered equivalent to formation displacement, with fiber optics undergoing only axial deformation; (b) The spacing between each measurement point on the fiber optic is typically 5 to 10 m, so the measured strain and strain rate represent the average values over this interval; (c) At time t, the number of fracture elements is m. Using the basic solution of displacement discontinuity, the displacement of each element is calculated. Summing the displacements of all elements provides the displacement at each measurement point on the fiber optic [38]:

(9) $$u_{f}=\sum_{i=1}^{M}\frac{1}{8\pi (1-\nu )}\left[2(1-\nu )I_{1}-I_{2}\right]w_{i}$$

In the equation, the specific forms of the kernel functions $I_{1}$ and $I_{2}$ are as follows [38]:

(10) $$\begin{array}{l} I_{1}=[-\arctan\left(\frac{\left(x-\xi )(y-\eta \right)}{rz}\right)]\mathrm{||}\\ I_{2}=\{\frac{z(x-\xi )(x-\eta )\left[{\left(x-\xi \right)}^{2}+r^{2}\right]}{r\left[{\left(y-\eta \right)}^{2}+{\left(x-\xi \right)}^{2}\right]\left[{\left(y-\eta \right)}^{2}+z^{2}\right]}\}\mathrm{||} \end{array}$$

where *r* and the operator ||, respectively, represent [38]:

(11) $$\begin{array}{l} r=\sqrt{{\left(x-\xi \right)}^{2}+{\left(y-\eta \right)}^{2}+z^{2}}\\ I(\xi ,\eta )\;\mathrm{||}=I(a,b)-I(a,-b)-I(-a,b)+I(-a,-b) \end{array}$$

where *a* = 0.5Δ*x* and *b* = 0.5Δ*y*.

Based on the central difference method and the relationship between displacement and strain, the axial strain at the measurement point can be calculated from the axial displacement at the measurement point, as follows:

(12) $$\varepsilon =\frac{u_{f}(z+L/2)-u_{f}(z-L/2)}{L}$$

It is noted that this expression represents the central difference approximation of the spatial derivative of fiber displacement with respect to the axial coordinate *z*. Since DAS measurements are taken over discrete intervals (typically 5–10 m), the derivative is approximated using this finite-difference form, which captures the average strain over the sensing gauge length. The strain rate can be calculated based on the strain at adjacent time points:

(13) $$\dot{\varepsilon }=\frac{\varepsilon (t+\Delta t)-\varepsilon (t)}{\Delta t}$$

When the displacement of the distributed optic fiber is continuous, the fiber displacement calculated using the axial strain formula equals the formation displacement. However, when the displacement of the distributed optic fiber is discontinuous (e.g., when fractures extend to the fiber), the displacement near the fracture surface becomes discontinuous. In such discontinuity regions, the fiber strain calculated from the fiber displacement differs from the formation strain, while the continuous regions still reflect the formation strain.

### 2.3. Inversion Algorithm

In this study, the inversion of fracture propagation is conducted using the PKN model, ensuring the accuracy of fracture geometry through full-time-step fitting. The key innovation of this approach lies in fitting the entire temporal sequence rather than relying on individual time points or localized data, which overcomes the limitations of conventional inversion methods. By capturing the complete evolution of fracture propagation, the proposed method ensures consistency and correctness in fracture morphology, thereby enhancing the reliability of the inversion results.

The inversion process is optimized by adjusting four key parameters. The first parameter is the fracture height, which governs the vertical extent of the fracture and significantly influences the shape of the waterfall plot. The second parameter is the displacement of the fracture along the height direction, representing spatial deviations and reflecting the relative positioning between the fracture and the fiber optic sensor. Adjusting this displacement enables better correction of fracture positioning, thereby improving the accuracy of model fitting. The third and fourth parameters are two scaling coefficients, *α_pos_* and *α_neg_*, which independently adjust the positive and negative strain rates in the fracture model. These coefficients are introduced to compensate for the effects of casing and other structural influences on the strain rate, particularly in regions where the strain rate exhibits positive or negative values. By fine-tuning these two parameters, the impact of casing-induced distortions is minimized, thereby enhancing the agreement between the inverted model and the measured strain data.

The PKN model parameters (such as fracture height *H* and half-length *L*) are directly involved in calculating the displacement and width of each fracture segment, which then drives the strain response on the fiber. In contrast, *α_pos_* and *α_neg_* do not affect the fracture geometry but instead serve as empirical correction factors to adjust the magnitude of the modeled strain rate based on its sign. These scaling parameters refine the match between model output and real DAS measurements after the mechanical deformation has been computed using the physical fracture model. Thus, the optimization updates H and offset to control the actual fracture geometry, while *α_pos_* and *α_neg_* provide signal-level calibration to account for systematic discrepancies between model assumptions and field measurements.

The optimization of the loss function is based on the fitting error of the strain rate. First, the predicted strain rate is computed, followed by an adjustment based on its sign. Specifically, a scaling factor *α_pos_* is applied to positive strain rates, while a separate factor *α*neg is applied to negative strain rates. The adjusted strain rate is formulated as follows:

(14) $$\varepsilon_{fitted}=\left\{\begin{array}{ll} \alpha_{pos}\cdot \varepsilon_{calculated}, & \mathrm{if}\;\varepsilon_{calculated}\geq 0\\ \alpha_{neg}\cdot \varepsilon_{calculated}, & \mathrm{if}\;\varepsilon_{calculated}\leq 0 \end{array}\right.$$

where *ε_fitted_* represents the adjusted strain rate after scaling, *ε_calculated_* is the initially computed strain rate from the model, *α_pos_* and *α_neg_* are the scaling coefficients for the positive and negative strain rates, respectively. These coefficients are iteratively optimized to minimize the discrepancy between the modeled and measured strain rates. The optimization of this loss function refines the parameters *α_pos_* and *α_neg_*, ensuring accurate inversion of fracture geometry and strain evolution.

Next, the loss function optimizes the model by quantifying the discrepancy between the adjusted strain rate and the measured strain rate (*ε_measured_*). Specifically, the loss function is defined as the Frobenius norm of the difference between the adjusted strain rate and the actual measured strain rate, representing the Euclidean distance between them:

(15) $$Loss={\left\|\varepsilon_{fitted}-\varepsilon_{measured}\right\|}_{F}$$

By optimizing this loss function, the parameters *α_pos_* and *α_neg_* are iteratively adjusted to minimize the deviation between the predicted and observed strain rates. This ensures that the model accurately captures the strain response, ultimately leading to a precise inversion of the fracture geometry.

During the inversion process, parameter optimization follows a carefully designed iterative procedure. At each iteration, the optimization progress is displayed in real-time, allowing researchers to monitor the convergence behavior and evaluate the effectiveness of parameter updates. To ensure that the optimization algorithm has sufficient computational resources and does not terminate prematurely, a high maximum iteration count and function evaluation limit are set. These settings prevent the optimization process from being hindered by computational constraints and enable the identification of a global optimal solution. Compared with traditional inversion methods that directly fit fiber optic responses by adjusting time-dependent fracture width parameters, our approach employs a full-time-step inversion constrained by the PKN model. This eliminates the need to impose additional constraints such as width symmetry, temporal smoothness, or predefined fracture width evolution. As a result, although our method still involves a certain computational cost, it significantly improves efficiency by reducing the number of free parameters. More importantly, the inversion process becomes more stable, controllable, and consistently convergent due to the underlying physics-informed framework. Additionally, stringent step tolerance and optimality tolerance thresholds are imposed to maintain numerical precision in parameter updates, thereby reducing the risk of instability due to computational errors. This is particularly critical when handling large computational loads and complex parameter interactions, as it ensures the robustness and reliability of the optimization process.

In summary, the inversion approach not only guarantees the accuracy of fracture geometry reconstruction through full-time-step fitting but also enhances model performance through precise parameter adjustments. This methodology maximizes the fidelity of the inversion results, ensuring both accuracy and consistency in capturing the dynamic evolution of the fracture.

## 3. Experiment and Result

To demonstrate the effectiveness of the proposed inversion framework, two types of data were used in this study: synthetic and field data. The synthetic data were generated using the PKN fracture model and the fiber optic strain response model to simulate DAS strain rate signals. These provide a controlled environment to verify the accuracy and stability of the inversion method. The field data were obtained from the Hydraulic Fracturing Test Site (HFTS), where DAS was used to monitor strain rate evolution during real hydraulic fracturing operations. These data enable us to test the applicability of the method under practical conditions.

In this study, we first simulated the fracture propagation process using the PKN model to ensure that the fracture geometry was accurately captured over time. To achieve this, we employed a full-time-step fitting approach to preserve the consistency of the fracture morphology. Based on the fiber optic strain response data, we generated waterfall plots of strain evolution during fracture propagation, providing a foundation for subsequent inversion tests. We then performed inversion on the simulated results using an idealized model to validate the accuracy and applicability of the proposed approach. Finally, the strain rate waterfall plots obtained from simulations were compared with field data from HFTS fiber optic measurements to further assess the effectiveness of the method in practical applications. Through these steps, we comprehensively demonstrate the potential of the inversion methodology for fiber optic-based near-wellbore fracture monitoring and provide valuable insights for future research.

### 3.1. Fiber Optic Strain Response

The forward fiber optic modeling serves as the foundation for fracture morphology inversion. Using the PKN model, we first simulated the fracture propagation process and derived strain waterfall plots from fiber optic strain response data. These forward simulations allow us to capture the spatiotemporal evolution of displacement, strain, and strain rate throughout fracture propagation, providing essential reference data for subsequent inversion analyses. In particular, we examine the spatial distribution of strain and strain rate at different fracture propagation stages, focusing on stress concentration regions and the corresponding strain variations.

**Figure 2:**
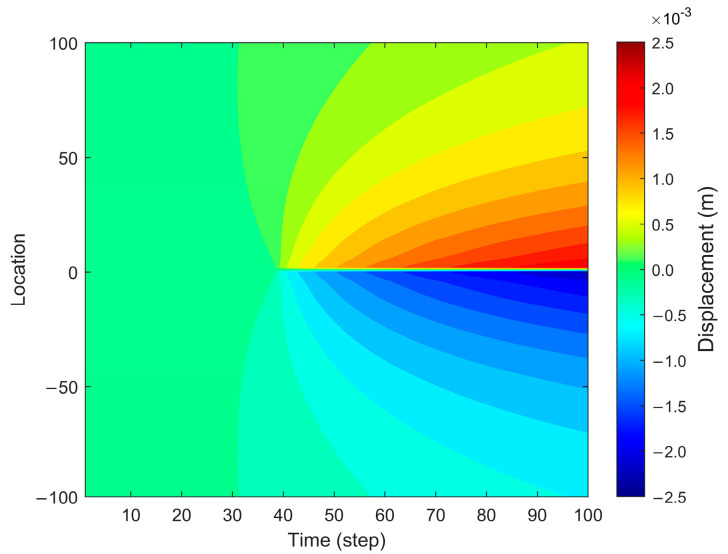
Displacement in the forward fiber optic model.

Figure 2 illustrates the displacement field in the forward fiber optic model, highlighting the fracture opening process along the fracture width direction. As fracture propagation progresses over time, the displacement expands outward from the initial fracture tip, forming a gradual spatial evolution pattern. The displacement distribution exhibits an increasing trend from the fracture center toward the outer regions, with displacement values significantly increasing as the fracture propagates.

**Figure 3:**
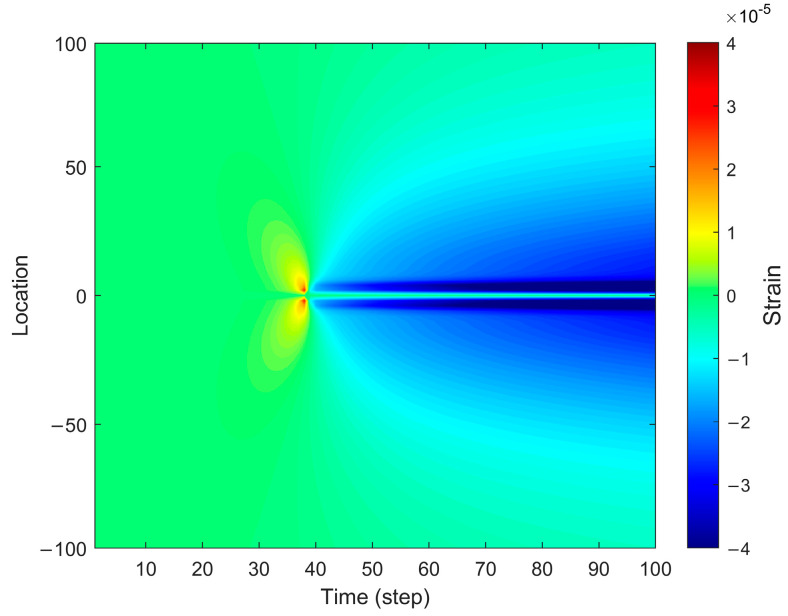
Strain distribution in the forward fiber optic model.

Figure 3 presents the strain distribution during fracture propagation, revealing distinct spatial characteristics. Notably, the strain values exhibit significant localization near the fracture tip, forming regions of concentrated strain. The color transitions in the figure clearly illustrate the temporal variation of strain, with strain values at the fracture tip and propagation front continuously increasing. This indicates that the influence of fracture propagation on the surrounding medium intensifies over time.

**Figure 4:**
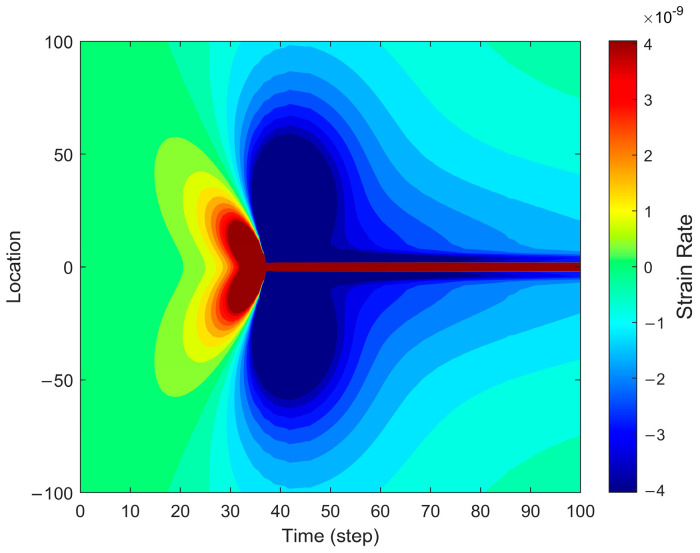
Strain rate distribution in the forward fiber optic model.

Figure 4 depicts the strain rate distribution during fracture propagation, capturing the dynamic characteristics of strain evolution. The strain rate exhibits peak values near the fracture tip, particularly in the opening region where the propagation rate increases sharply. This phenomenon arises from the rapid stress redistribution induced by fracture expansion, leading to an abrupt increase in local strain rate. As the fracture continues to propagate, the strain rate distribution gradually extends away from the fracture tip, displaying a noticeable attenuation trend. Additionally, the figure highlights the spatial distribution of strain rate, showing that the highest strain rate values are concentrated near the fracture tip, whereas regions farther from the fracture exhibit significantly lower strain rates. This spatial variation indicates the heterogeneous nature of strain rate distribution during fracture propagation, where the fracture tip represents the most active zone, while distant regions experience minimal strain rate variations.

### 3.2. Inversion Testing with an Idealized Model

In this section, we present the inversion test results obtained using an idealized model and compare them with actual fracture data and the results obtained from full-time-step inversion based on the PKN model. By analyzing the variations in strain rate, fracture half-length, and fracture width, we validate the effectiveness and accuracy of the PKN model in fracture propagation simulation. Specifically, the full-time-step fitting method employed in this study ensures the accuracy of the fracture morphology and overcomes the limitations of traditional single-time-step inversion methods. By comparing the measured data with the computed results, we comprehensively assess the applicability and performance of the inversion method in reconstructing fracture propagation and provide strong support for its future applications. The following section presents three sets of comparison figures: single-time-step strain rate comparison, fracture half-length comparison, and fracture width comparison, to further evaluate the effectiveness of the inversion tests.

**Figure 5:**
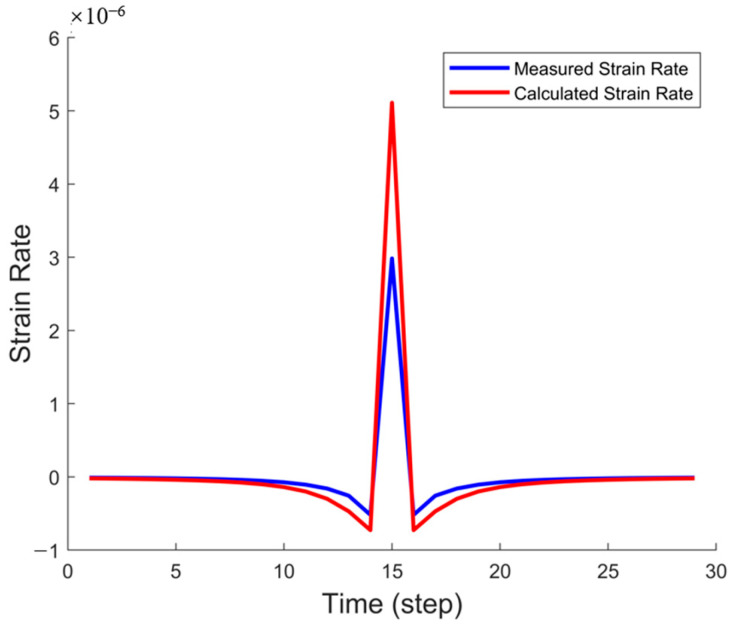
Single-time-step strain rate comparison in the idealized model.

Figure 5 illustrates the comparison between the measured and computed strain rates at a specific time step. The blue curve represents the actual strain rate data obtained from fiber optic monitoring, while the red curve corresponds to the strain rate inverted using the PKN model. The results indicate a strong agreement between the two across most time steps, particularly during the rapid fracture propagation phase and in regions where the strain rate exhibits negative values. This confirms that employing full-time-step fitting with the PKN model effectively captures the evolution of strain rate during fracture propagation, thereby validating the accuracy and reliability of the full-time-step inversion approach.

**Figure 6:**
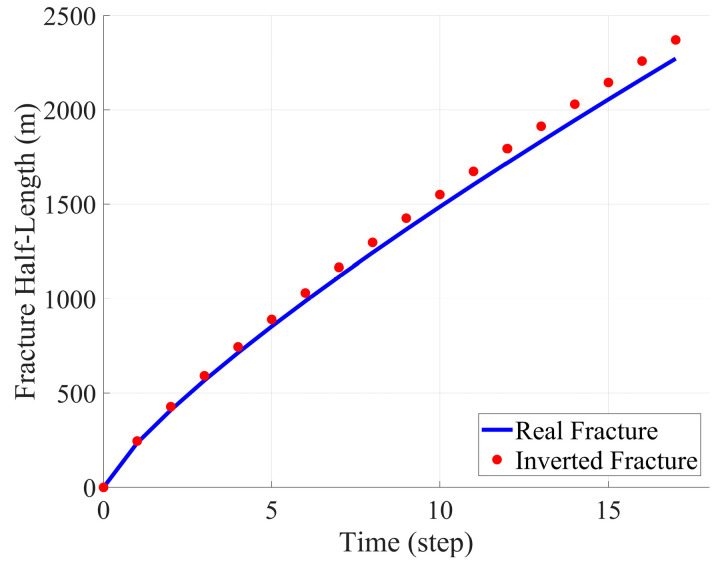
Comparison of fracture half-length between real fracture and inversion fracture.

Figure 6 further illustrates the temporal evolution of the fracture half-length. The blue curve represents the actual fracture propagation trajectory, while the red markers denote the fracture half-length obtained using the full-time-step inversion method. The figure shows a strong correlation between the two trends, particularly in the early stages of fracture propagation, where the propagation velocity is nearly identical. The full-time-step inversion approach successfully reconstructs the fracture propagation process, demonstrating the high accuracy of the PKN model in capturing fracture geometry.

**Figure 7:**
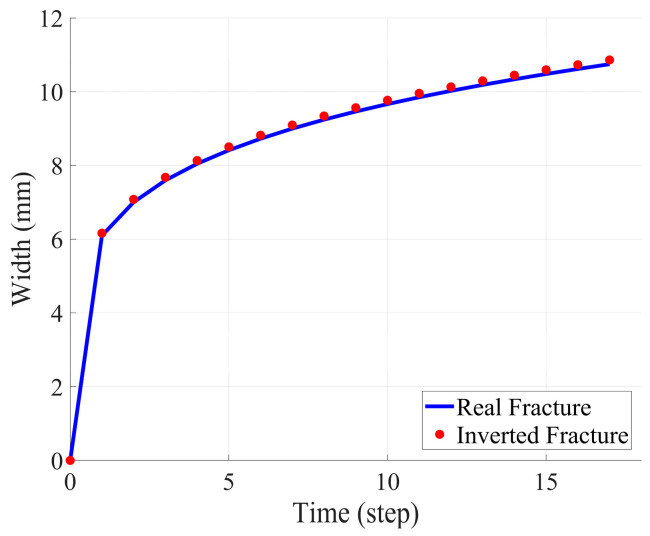
Comparison of crack widths between real cracks and inversion cracks.

Figure 7 presents the variations in fracture width at different time steps. Similar to the previous figures, the blue curve represents the actual fracture width evolution, while the red markers indicate the fracture width obtained through inversion. The results show that the fracture width variation aligns closely with the actual measurements, confirming that the PKN model effectively reproduces fracture width evolution during propagation.

Through these comparisons, we validate the effectiveness of the full-time-step inversion approach based on the PKN model. This method ensures the accurate reconstruction of fracture morphology and overcomes the potential errors and limitations associated with traditional single-time-step inversion techniques. By employing full-time-step inversion, we not only enhance the accuracy of the fracture geometry reconstruction but also capture the spatiotemporal dynamics of fracture propagation more comprehensively, providing reliable support for practical applications.

### 3.3. Field Data Inversion from HFTS

In this section, we compare the fiber optic strain waterfall plots obtained from HFTS field monitoring with the results computed using the idealized model. This comparison allows us to evaluate the performance of the inversion method in real-world applications and further assess the accuracy of the PKN model in fracture propagation simulation.

**Figure 8:**
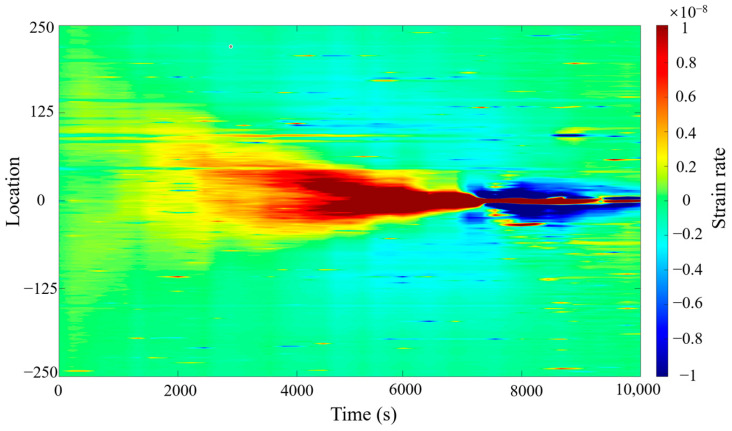
Strain rate waterfall plot from HFTS field data.

Figure 8 depicts the strain rate variations recorded during fracture propagation at the HFTS site using fiber optic monitoring. The figure illustrates the dynamic evolution of strain rate as it propagates outward from the fiber optic sensor location during fracture growth. As the fracture extends, the strain rate exhibits rapid changes near the fracture tip, demonstrating a distinct asymmetry. This variation in strain rate is closely linked to the fracture geometry and propagation velocity, reflecting the characteristics of stress concentration and dynamic fracture evolution.

**Figure 9:**
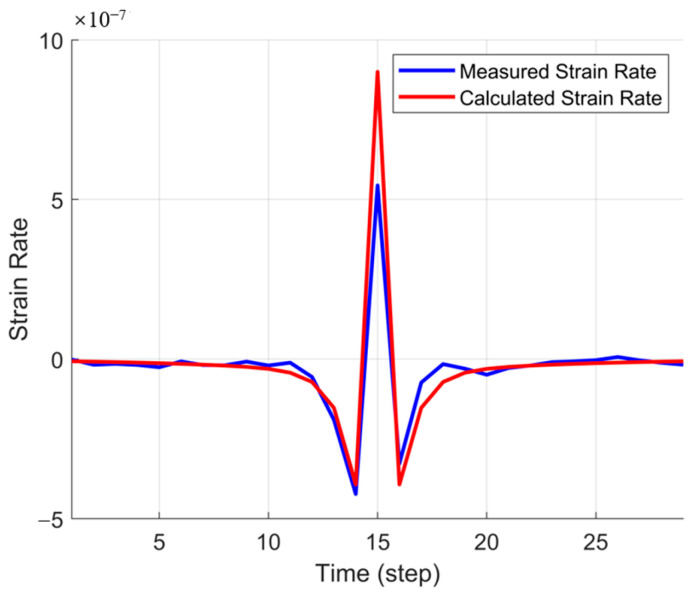
Comparison of strain rates in a single time step.

Figure 9 presents a comparison of the strain rate at a specific time step between the HFTS field data and the computed results from the PKN model. The blue curve represents the measured strain rate, while the red curve denotes the computed strain rate. The two datasets exhibit a strong correlation, indicating that the PKN model effectively captures strain rate evolution during fracture propagation. Although minor deviations are observed near peak values, the overall agreement between the two confirms the accuracy and applicability of the inversion method.

**Figure 10:**
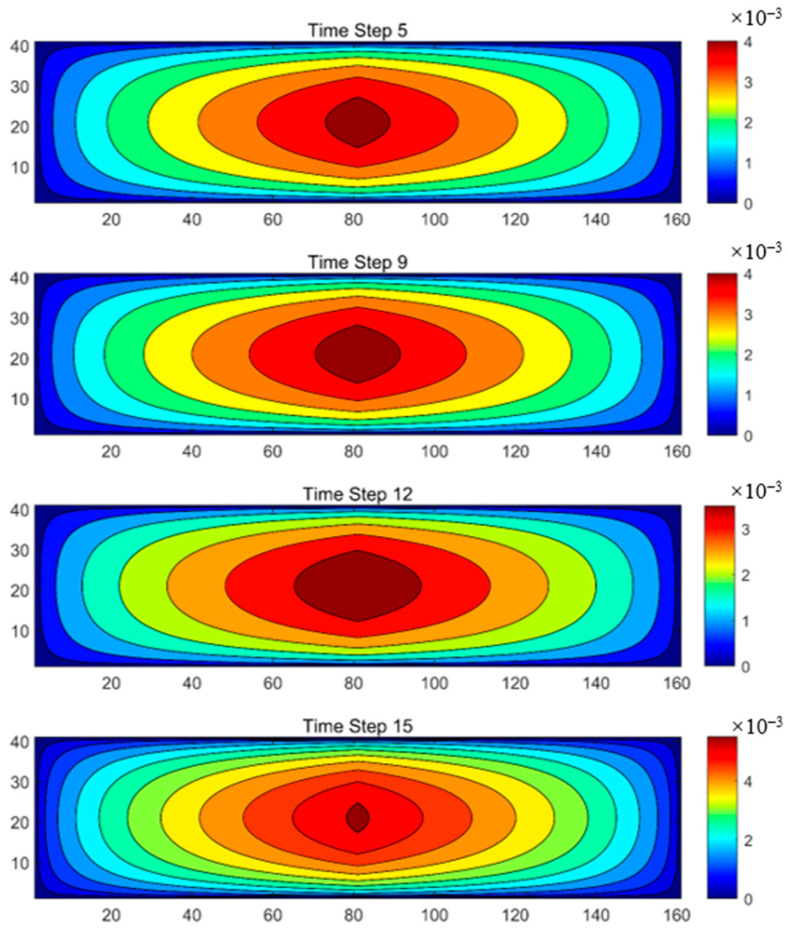
Inverted fracture morphology.

Figure 10 illustrates the fracture propagation at four different time steps, capturing the spatial distribution of strain during fracture evolution. As time progresses, the fracture expansion region gradually enlarges. In the figure, the red areas represent the primary fracture propagation zones, while the blue areas indicate regions unaffected by fracture growth. These images provide a clear visualization of the fracture propagation patterns and the temporal evolution of fracture geometry. The results confirm that our inversion method successfully reconstructs fracture propagation dynamics.

Through the analysis of these images, we further validate the effectiveness of the PKN model and the inversion approach in real-world applications, demonstrating their potential for fracture monitoring in practical engineering scenarios.

### 3.4. CPU Times

To further evaluate the computational efficiency of the proposed method, we compared its CPU time with that of a conventional inversion strategy. Although our method involves iterative optimization over all time steps, its computational structure is fundamentally different from traditional approaches.

Traditional fracture inversion methods typically treat fracture width as an explicit function of both space and time and optimize its evolution to fit fiber optic signals. To stabilize the inversion, additional constraints such as smoothing in time, symmetry enforcement in space, or empirical width evolution models are often applied. These lead to a high-dimensional optimization problem involving a large number of parameters across the fracture grid and time steps, significantly increasing computational costs.

In contrast, our method uses a full-time-step inversion framework based on the PKN model. Instead of fitting the full fracture width field, the method fits only a small set of physically meaningful parameters that control the overall fracture propagation and geometry. This leads to an implicit but physically constrained reconstruction of the fracture width. Essentially, it reduces the dimensionality of the inversion problem from a space-time distributed field to a handful of model parameters, resulting in much lower computational complexity and more stable convergence behavior.

**Figure 11:**
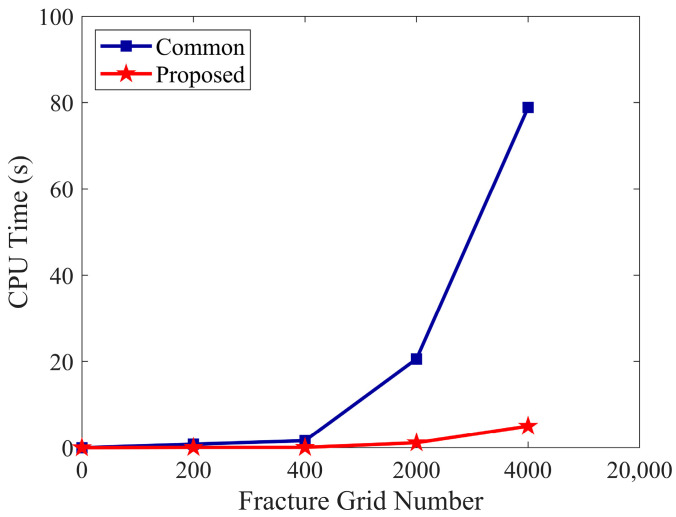
Comparison of CPU time between the proposed inversion method and a conventional fracture width-based approach under increasing fracture grid numbers.

As shown in Figure 11, the CPU time of the proposed method grows much more gently with increasing fracture grid resolution, while the conventional method scales poorly due to its parameter explosion.

## 4. Conclusions

In this study, we proposed a full-time-step fracture inversion method guided by the classical PKN model and strain rate response mechanisms of distributed acoustic sensing (DAS). The method was first validated using synthetic data generated from idealized fracture propagation, and then applied to real field DAS measurements from the Hydraulic Fracturing Test Site. The results show that the reconstructed fracture morphology and strain rate response align well with both synthetic and field data, confirming the effectiveness and robustness of the inversion framework.

As an advanced real-time monitoring technique, optic fiber downhole sensing has been widely applied in monitoring fracture propagation during hydraulic fracturing. However, existing fracture shape inversion methods face two main challenges: firstly, traditional methods struggle to accurately capture the dynamic changes in strain rate and fracture shape during the propagation process, and secondly, they are computationally expensive. To address these issues, this study proposes a full-time-step fitting inversion method based on the Perkins-Kern-Nordgren model. By precisely fitting each time step of fracture propagation, this method effectively overcomes the shape deviation problems often encountered in traditional methods and significantly reduces computational costs. Compared to conventional single-time-step inversion methods, our approach not only provides a more accurate representation of the spatiotemporal dynamics of fracture propagation but also avoids the risk of significant errors in fracture shape reconstruction. Therefore, the proposed inversion method holds substantial practical value and significance in fracture monitoring and assessment for oil and gas fields.

Overall, this study demonstrates the feasibility and reliability of using full-time-step inversion techniques for DAS-based fracture characterization. The method provides a strong foundation for advancing near-wellbore monitoring technology in unconventional reservoirs and has potential applications in other domains involving distributed fiber optic sensing.

In the future, this inversion framework could be further enhanced by integrating machine learning techniques, such as data-driven feature recognition or fracture pattern prediction, to improve automation and adaptability in complex geological settings. This direction holds great promise for real-time fracture monitoring and decision-making in broader fiber optic sensing applications.

## Data Availability

The original contributions presented in this study are included in this article. Further inquiries can be directed to the corresponding author.
